# Transabdominal Preperitoneal (TAPP) Approach for Spigelian Hernia: Our Clinical Experience and a Comprehensive Review of the Literature

**DOI:** 10.7759/cureus.94856

**Published:** 2025-10-18

**Authors:** Kristo Qylafi, Helena Hanschell, Anang Pangeni, Roland Fernandes, Sanjoy Basu, Ashish Shrestha

**Affiliations:** 1 General Surgery, Kent, Surrey and Sussex Deanery, Ashford, GBR; 2 General Surgery, William Harvey Hospital, East Kent Hospitals University NHS Foundation Trust (EKHUFT), Ashford, GBR; 3 Surgery, East Kent Hospitals University NHS Foundation Trust (EKHUFT), Ashford, GBR; 4 General Surgery, East Kent Hospitals University NHS Foundation Trust (EKHUFT), Ashford, GBR

**Keywords:** incarcerated spigelian hernia, spigelian aponeurosis, spigelian hernia, spigelian line, transabdominal preperitoneal (tapp)

## Abstract

Introduction

Spigelian hernia is a rare but clinically significant entity due to its high risk of incarceration, requiring prompt surgical intervention. Historically, the open approach was the preferred option; however, the current trend has shifted towards laparoscopic techniques. The transabdominal preperitoneal (TAPP) approach offers the advantage of satisfactory exposure with minimal invasion. This study aimed to review the management of Spigelian hernias and compare this with an institutional experience of surgical repair.

Methodology

A retrospective analysis was undertaken of prospectively collected data from all patients who underwent repair of Spigelian hernias at a large District General Hospital (DGH) between 2018 and 2024. Data included patient demographics, diagnostic investigations, operative details, length of hospital stay, follow-up and recurrence.

Results

The literature supports a laparoscopic approach, which is associated with fewer complications and shorter hospital stays compared to open repair. The institutional series included nine patients (male-to-female ratio, 4:5; median age, 75 years; body mass index (BMI), 24; and the American Society of Anesthesiologists (ASA) physical status class III). All patients underwent TAPP repair. There were no intraoperative complications, and the median hospital stay was one day. At a 12-month follow-up, there were no recurrences and one seroma, which was managed conservatively.

Conclusions

TAPP repair appears to be a safe and effective approach for the treatment of Spigelian hernias. While these findings align with the existing literature, further studies with larger sample sizes are needed to confirm long-term outcomes.

## Introduction

Spigelian hernia is a rare abdominal wall hernia that occurs when a sac protrudes through a defect in Spigelian's aponeurosis, first described by the 17th-century anatomist Adrian van der Spiegel. This defect lies in the transversus abdominis aponeurosis, between the lateral border of the rectus abdominis muscle and the semilunar line (now described as *EIT ambivium*) [1-8]. Joseph Klingosh was the first to formally describe the Spigelian hernia as a distinct clinical entity. Initial reports solely attributed the condition to trauma [6,9]; however, it is now recognised that Spigelian hernias may also develop spontaneously [3]. 

Although spigelian hernias may occur anywhere along the Spigelian fascia, the majority are found below the arcuate line. This region is particularly susceptible due to the absence of the posterior rectus sheath and the parallel orientation of the fascial fibres, both of which contribute to the localised weakness [5]. 

Despite its rarity, Spigelian hernia is clinically significant because of its high complication rates, which include incarceration, obstruction and strangulation - a risk accentuated by the extremely narrow hernial neck [1-2]. Reported risks of incarceration range between 17% and 30%, necessitating prompt surgical intervention [2-4]. Traditionally, open repair was preferred, but with advances in laparoscopic techniques, the trend has shifted to minimally invasive approaches as the intervention of choice [1,5]. 

This study aimed to evaluate the safety and efficacy of laparoscopic transabdominal preperitoneal (TAPP) repair for Spigelian hernia by comparing our institutional experience with outcomes reported in the published literature.

## Materials and methods

This was a retrospective analysis of prospectively collected data of all Spigelian hernias that were surgically managed between 2018 and 2024 in a large District General Hospital (DGH). Patient demographics, investigations (computed tomography), and operative details, including complications, hospital stay and follow-up, were collected and analysed. Both elective and emergency cases were included. Every patient underwent pre-operative computed tomography (CT) scan to confirm the diagnosis and delineate defect size. All patients attended a face-to-face follow-up at eight weeks, followed by a telephone review at 12 months. Finally, a literature search was conducted in PubMed, in accordance with the Preferred Reporting Items for Systematic Reviews and Meta-Analyses (PRISMA) 2020 guidelines [10].

Surgical technique

Under general anaesthesia with pre-operative prophylactic antibiotics, patients were positioned supine in a 10-degree reverse Trendelenburg position, tilted 10 degrees on the side of the hernia. Pneumoperitoneum was established using a Veress needle, and a 12 mm bladeless port 5 cm lateral to the umbilicus (opposite to the side of the hernia) was inserted. Subsequently, one 10 mm and another 5mm bladeless port were introduced at a distance of 8 cm on either side of the camera port (same horizontal line), under direct vision to minimise the risk of visceral/vascular injury. A 30-degree camera was used for the procedure. 

**Figure 1:**
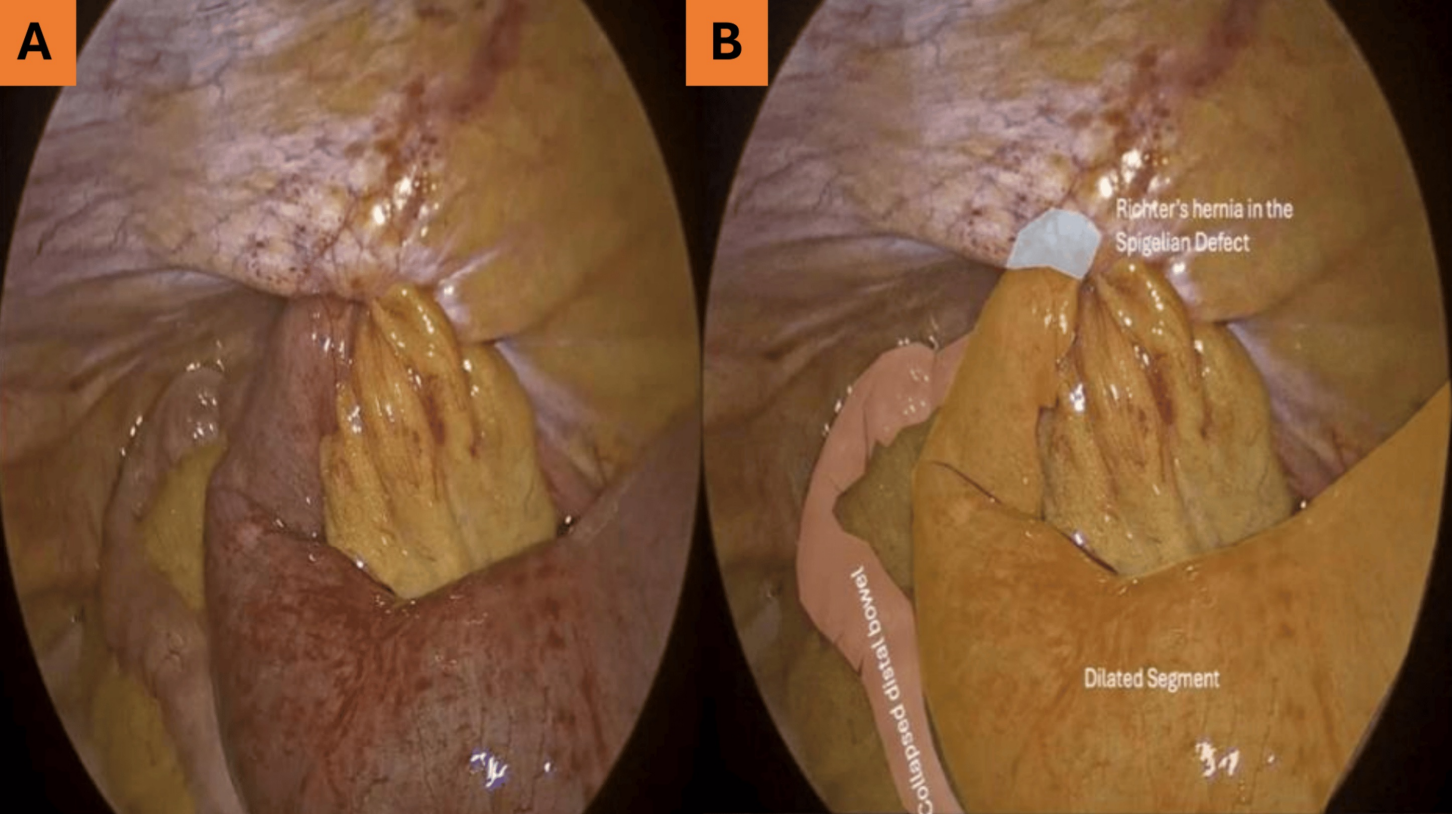
Hernia identification. (A) Intraoperative view showing the hernia sac protruding through the Spigelian defect. (B) Richter’s hernia within the Spigelian defect, involving a dilated segment of bowel.

**Figure 2:**
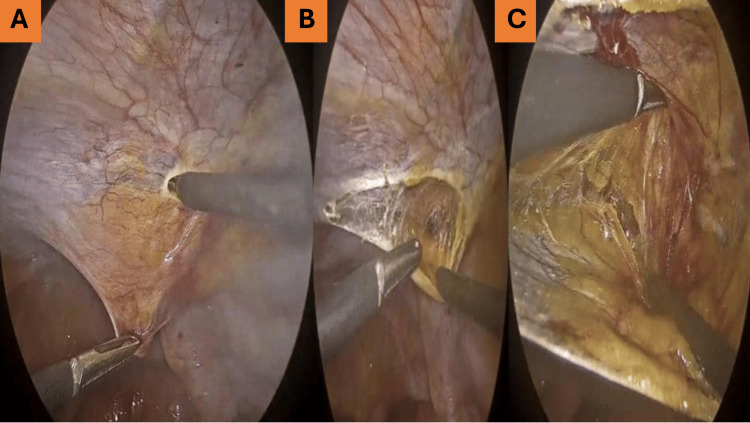
Incision of the parietal layer followed by sharp dissection to create a peritoneal flap. (A) Initial incision of the parietal peritoneum. (B) Sharp dissection to create a peritoneal flap. (C) Development of the preperitoneal space.

**Figure 3:**
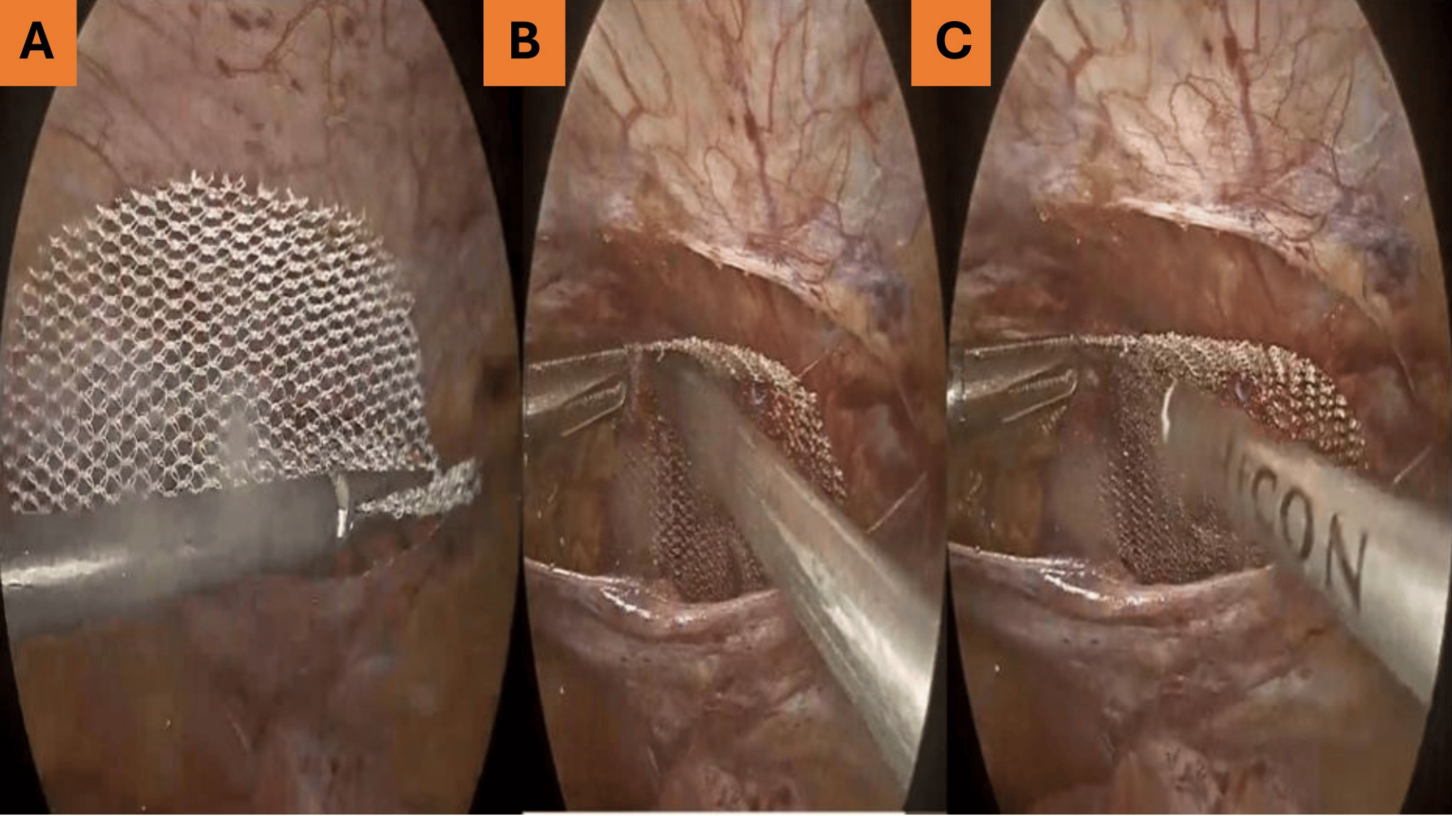
Mesh fixation using a plain polypropylene 10 × 7 cm mesh. (A) Introduction of a plain polypropylene 10 × 7 cm mesh. (B) Placement of the mesh with adequate overlap. (C) Fixation of the mesh to the abdominal wall using tackers.

**Figure 4:**
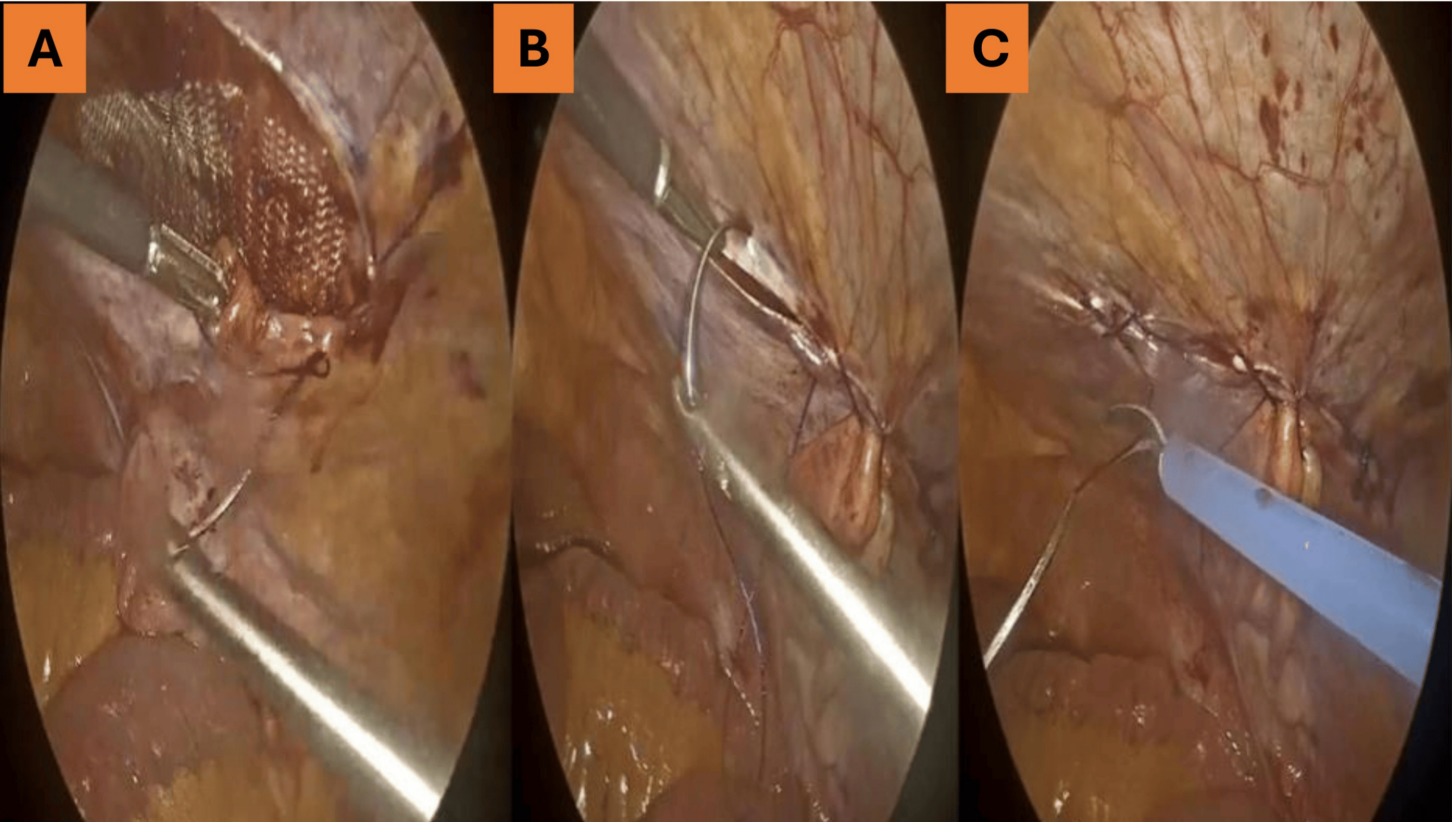
Defect closure with V-Loc suture. (A) Mesh fully positioned over the defect. (B) Closure of the peritoneal flap using a continuous V-Loc suture. (C) Complete closure with the mesh fully covered.

Hernia contents were identified and reduced (Figure 1). The peritoneum was incised 5 cm medial to the defect, and diathermy dissection was performed to create the peritoneal flap and the preperitoneal space (Figure 2). The hernia sac was identified and reduced. The defect was then measured and closed primarily using a continuous 2-0 V-Loc suture (Medtronic, Dublin, Ireland). An appropriate size (based on the original defect size plus a 5 cm overlap) synthetic mesh was inserted and fixed to the anterior abdominal wall through four corner stitches and tackers (Figure 3). Finally, the peritoneal flap was brought in front to cover the synthetic mesh and closed with continuous 2-0 V-Loc suture (Figure 4).

Haemostasis was confirmed, pneumoperitoneum was deflated under direct vision and the 10-mm port sites were closed with absorbable material.

## Results

The study included nine patients, with a male-to-female ratio of 4:5, a median age of 75 years (range 48-88), a body mass index (BMI) of 24 (range 20.07-41.78) kg/m^2^, and the American Society of Anesthesiologists (ASA) physical status grade III. Patient demographics and operative details are summarised in Table 1.

**Table 1:** Our departmental series. BMI, body mass index; ASA, American Society of Anesthesiologists

Patient number	Age	Sex	BMI (kg/m^2^)	ASA	Type of admission	Defect size (cm)	Procedure	Complications	Length of stay (days)	Follow-up
1	61	M	41.78	III	Elective	5	TAPP	Seroma	2	No recurrence
2	76	M	30.79	III	Elective	3	TAPP	Nil	1	No recurrence
3	88	M	30.71	III	Emergency	2.2	TAPP	Nil	3	No recurrence
4	78	F	23.40	III	Elective	2.3	TAPP	Nil	0	No recurrence
5	75	F	23.42	II	Elective	3.1	TAPP	Nil	0	No recurrence
6	63	F	23.87	I	Elective	3.6	TAPP	Nil	1	No recurrence
7	71	M	20.07	III	Elective	4.4	TAPP	Nil	1	No recurrence
8	79	F	27.93	III	Elective	2.9	TAPP	Nil	0	No recurrence
9	48	F	24	II	Elective	2	TAPP	Nil	1	No recurrence

All patients underwent laparoscopic TAPP repair. The mean operative time was 82 (range 60-120) minutes. No intraoperative complications were recorded. The mean length of hospital stay was 1 day (range 0-3). There were no conversions to open surgery. Only one patient developed a seroma, which was treated conservatively. At 12-month follow-up, there were no recurrences.

## Discussion

**Figure 5:**
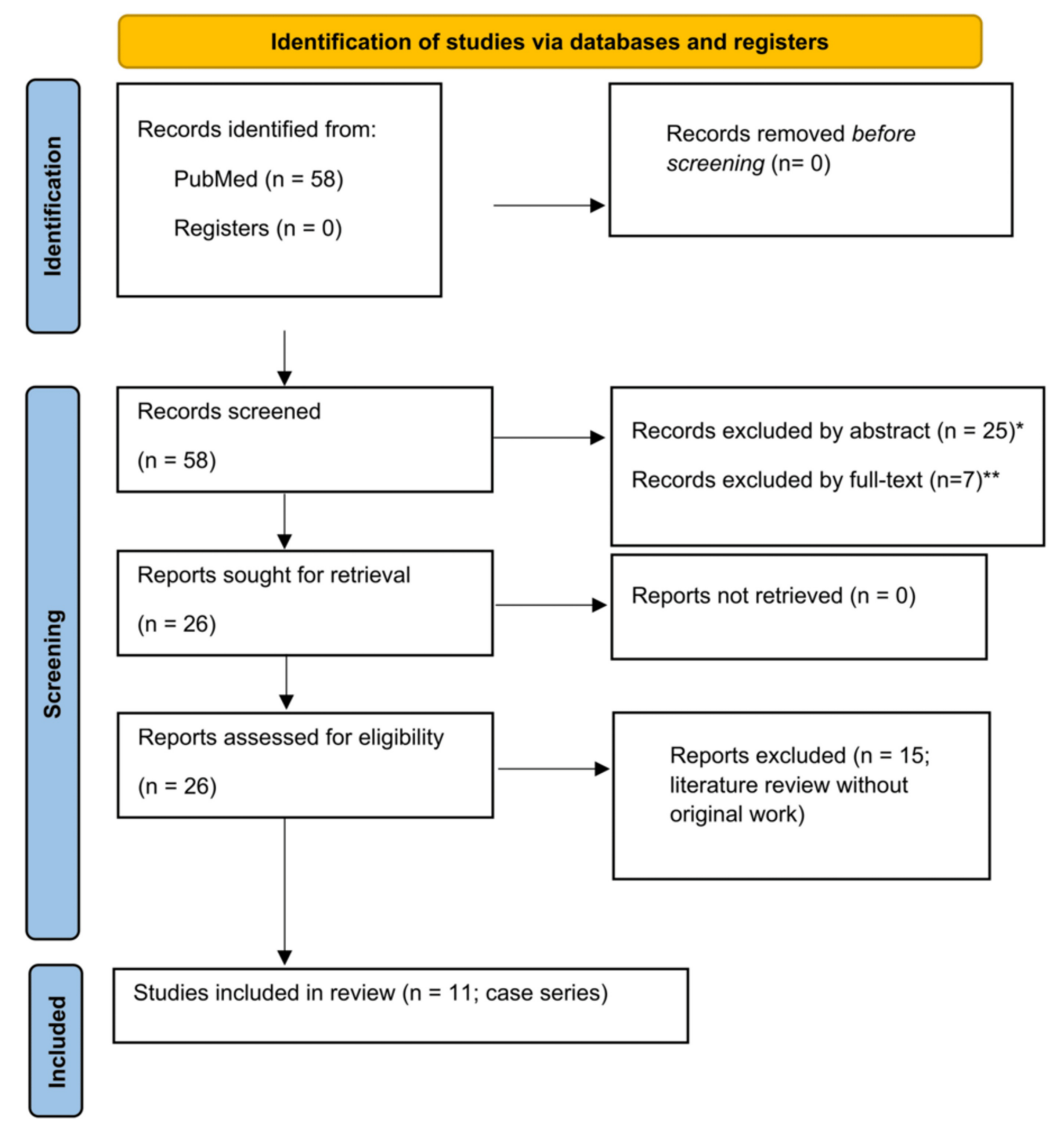
PRISMA flow diagram. ^*^Reason for exclusion: Non-English publications (*n* = 6), paediatric population (*n* = 15), and irrelevant topic (*n* = 4). ^**^Reason for exclusion: Single case reports (*n* = 5), irrelevant topic (*n* = 2). PRISMA, Preferred Reporting Items for Systematic Reviews and Meta-Analyses

A structured literature search was conducted in PubMed using the terms "Spigelian hernia AND laparoscopic repair". The inclusion criteria comprised English-language articles reporting original clinical data on adult patients undergoing surgical repair of Spigelian hernia (either open or laparoscopic). Exclusion criteria included single-case reports, paediatric cases and non-English publications. The study selection process is presented in Figure 5. After the removal of irrelevant papers, 58 articles were screened. Of these, 32 were excluded. The remaining 26 full-text articles were assessed for eligibility. Fifteen were literature reviews and 11 were case series. Only the case series are summarised in Table 2 [4-25], as they presented original clinical data. The literature review identified open repair as the most widely used technique, with laparoscopic Intraperitoneal Onlay Mesh (IPOM) repair being performed in 45% cases [11,12]. The totally extraperitoneal (TEP) approach offers the advantage of keeping the mesh completely outside the peritoneal cavity, thereby reducing exposure to the abdominal viscera and related complications. However, technically, it is more challenging and has a steeper learning curve [8]. Seroma and haematoma were reported as the most frequent complications, while wound infection and conversion to open repair were less common. No recurrences were identified in the reviewed literature [8,11,12].

**Table 2:** Summary of studies reviewed. SILTEP, single-incision laparoscopic totally extraperitoneal; TEP, totally extraperitoneal; IPOM, intraperitoneal onlay mesh; TAPP, transabdominal preperitoneal

Author	Type	Year	Number of patients	Technique	Mean hospital stay (days)	Complications	Recurrence
patients	Retrospective study	2004	2	Open repair (sutures)	-	Nil	Nil
Cui et al. [8]	Case series	2021	7	Laparoscopic repair (TEP)	1.4	1 seroma	Nil
Cinar et al. [11]	Retrospective study	2013	9	Open repair and laparoscopic repair	Open repair: 5.1; laparoscopic repair: 2.5	Nil	1
Moreno-Egea et al. [15]	Prospective RCT	2002	22	Open repair and laparoscopic repair	Open repair: 5.2; laparoscopic repair: 1.4	Open repair: 4 (haematoma); laparoscopic repair: 0	Nil
Leff et al. [16]	Retrospective study	2009	2	Laparoscopic repair (sutures)	1.5	Nil	Nil
Bittner et al. [17]	Retrospective study	2008	2	Laparoscopic repair (sutures)	-	Nil	Nil
Saber et al. [18]	Retrospective study	2008	8	Laparoscopic repair (scroll)	-	Nil	Nil
Moreno-Egea et al. [19]	Prospective analysis	2015	16	Laparoscopic repair (IPOM: 9; TEP: 7)	1	Nil	Nil
Patle et al. [23]	Prospective analysis	2010	6	Laparoscopic repair (TAPP)	1.5	1 seroma	Nil
Malazgirt et al. [24]	Prospective analysis	2006	34	Open repair and laparoscopic repair	Open repair: 6.5; laparoscopic repair: 1.7	5 patients: 3 seromas, 1 wound infection, 1 severe pain	Nil
Tran et al. [25]	Prospective analysis	2015	7	Laparoscopic repair (SILTEP)	0	1 haematoma	Nil

Spigelian hernias account for 0.12%-2% of all abdominal wall hernias, with the peak incidence between 50 and 60 years of age. Controversy exists regarding gender distribution, as certain studies have reported a higher female incidence, whilst others have found no significant difference [2-5]. In the present series, 5 patients were female and 4 were male. 

Spigelian hernias occur in the anatomical area bounded laterally by the semilunar line and medially by the lateral border of the rectus sheath. They are classified as high or low based on their relation to the inferior epigastric vessels. The contents of the hernial sac vary: most commonly omentum, small, or the large bowel, though, less frequently, other structures such as the testis or ovary have also been described [1-4]. 

Several theories have been proposed to explain the occurrence of Spigelian hernias at this particular anatomical area. The vascular-nervous theory states that hernias develop through defects in the abdominal wall caused by penetrating neurovascular bundles. The musculo-aponeurotic fasciculation theory, which is the most widely accepted, suggests that weak areas exist in the internal oblique and transversus abdominis aponeuroses with fibro-adipose deposition, through which the hernias protrude. Above the umbilicus, the fibres of the external oblique and transversus abdominis muscles run perpendicular to each other, providing strength to the abdominal wall. Below the umbilicus, however, the fibres of the two muscles run parallel, creating areas of weakness. Additional predisposing factors include obesity, chronic obstructive pulmonary disease (COPD), previous pregnancies and abdominal wall scars. Among reported cases, 50% of patients have undergone previous abdominal surgery [2,5]. Interestingly, none of the patients in this series had these risk factors apart from a high BMI.

Spigelian hernias most often present as a palpable mass at the defect site, with patients commonly reporting vague, non-specific abdominal pain. In the majority of cases, however, the sac lies beneath the external oblique-hence the eponym 'intraparietal’, resulting in minimal physical findings. Although some authors suggest that diagnosis can be made clinically, imaging, such as ultrasound (US) and Computed Tomography (CT) scan, is almost always required for confirmation. A CT scan has the advantage of being an operator-independent imaging modality and can delineate the anatomy while identifying additional pathology. Ultrasound, however, provides a radiation-free alternative that allows for dynamic assessment [1-5]. 

Spigelian hernias require surgical management due to their high risk of strangulation. However, because of its rarity, the available literature is limited to case reports and case series. Consequently, even the European Hernia Society (EHS) guidelines could not reach a consensus on the optimal surgical technique [13,14]. Traditionally, open surgery was the procedure of choice: the midline approach was preferred for the strangulated hernias as it provided better visualisation of the abdominal cavity and allowed exploration of the contralateral side, while the lateral approach was generally reserved for uncomplicated cases. With respect to mesh use, primary repair with sutures has been described as feasible [4,15,16]; however, numerous studies have reported high recurrence rates as high as 14-40%. Consequently, mesh reinforcement is considered the standard of care [2,3,5,16,17]. 

Laparoscopic repair of Spigelian hernias was first reported in 1992 [1]. Three techniques are currently practised: IPOM, TAPP and TEP repairs. Among these, IPOM is the most widely reported, accounting for 45% laparoscopic cases. A variation of IPOM, the *Scroll* technique, involves fixing the mesh to the abdominal wall before unfolding, thereby improving procedural efficiency [18]. The main drawback of IPOM is the risk of bowel adhesions, which can be mitigated by the TAPP approach; hence, the second most performed technique for Spigelian hernia repairs. TEP avoids entry into the abdominal cavity and thus reduces the risk of intra-abdominal injury. However, it is technically more challenging, providing limited space for dissection and is less frequently performed [8,9,11,15,19].

Moreno-Egea et al., in the only known randomised controlled trial (RCT), found no difference in recurrence rates between open and laparoscopic repair. However, several studies have reported lower morbidity and shorter hospital stays following laparoscopic repair [9,16,20-24]. Tran et al., in their series of seven patients, demonstrated the safety and effectiveness of a single-incision laparoscopic totally extraperitoneal (SILTEP) repair, which minimises abdominal wall trauma while allowing direct (telescopic) extraperitoneal dissection [25]. Kirkpatrick et al. reported the use of robotics for Spigelian hernia repair [26]. 

In the present series, all patients underwent TAPP repair with mesh placement. Recovery was uneventful, with a short hospital stay and no recurrence at 12-month follow-up.

Limitations

The study has several limitations. We acknowledge that the small number of patients restricts the statistical power of the findings. In addition, the study was conducted at a single centre, which may introduce surgeon-related bias. Another limitation is that patient-reported outcomes such as postoperative pain, quality of life and return to normal activity were not assessed. The 12-month follow-up period may also be insufficient to identify late recurrences or mesh-related complications. Finally, the retrospective design, although based on prospectively collected data, is limited by the absence of randomisation. 

## Conclusions

Spigelian hernia remains a rare but clinically significant entity due to its high risk of incarceration and strangulation. In this study, a series of nine patients were successfully treated via the TAPP approach, with no recurrence on follow-up. The literature review further supports the safety and efficacy of laparoscopy in comparison with open repair. Although larger multicentre studies are needed, these findings highlight that TAPP repair is a safe and effective technique for the management of Spigelian hernias.
